# Bleb Morphology on Anterior-Segment Optical Coherence Tomography after XEN Gel Stent Implantation

**DOI:** 10.3390/jcm12216740

**Published:** 2023-10-25

**Authors:** Seoyoung Wy, Young In Shin, Young Kook Kim, Jin Wook Jeoung, Ki Ho Park

**Affiliations:** 1 Department of Ophthalmology, Seoul National University College of Medicine, Seoul 03080, Republic of Korea; wsy445@gmail.com (S.W.); poohsyi@hanmail.net (Y.I.S.); eyedry@snu.ac.kr (Y.K.K.); neuroprotect@snu.ac.kr (J.W.J.); 2 Hangil Eye Hospital, Incheon 21388, Republic of Korea; 3 Department of Ophthalmology, Seoul National University Hospital, Seoul 03080, Republic of Korea

**Keywords:** XEN gel stent implantation, bleb morphology, anterior-segment optical coherence tomography

## Abstract

We investigated the internal morphology of filtration blebs after XEN gel stent implantation using anterior segment optical coherence tomography (AS-OCT) and identified factors related to intraocular pressure (IOP) postoperatively. Eighteen eyes of 18 patients who had undergone XEN gel stent implantation were analyzed. Blebs were imaged using Visante OCT (Carl Zeiss Meditec AG, Germany) at 6 months and 1 year after surgery and evaluated for quantitative parameters including bleb height, maximum height of internal cavity, maximum bleb wall thickness, and maximum bleb epithelial thickness. Subjects were classified into two groups according to the presence or absence of a definite internal cavity between the conjunctiva and sclera using AS-OCT imaging. Nine eyes (50%) were assigned to the internal cavity group and 9 (50%) to the uniform group. Postoperative IOP was significantly lower in the internal cavity group than in the uniform group both at 6 months and 1 year after surgery (*p* = 0.024 and *p* = 0.040). Postoperative IOP showed statistically significant negative correlations with bleb height and the height of the internal cavity (Spearman correlation coefficient r = −0.518, *p* = 0.028 and r = −0.453, *p* = 0.034, respectively). AS-OCT facilitates analysis of bleb morphology after XEN gel stent implantation. A larger height of the internal cavity of the bleb appeared to correlate with lower IOP after XEN implantation.

## 1. Introduction

Minimally invasive glaucoma surgeries (MIGS) are safer and less traumatic surgical interventions compared with conventional glaucoma surgeries, and are characterized by a good safety profile, minimal disruption of the normal conjunctival or scleral anatomy, rapid recovery for patients, and ease of use for surgeons [1]. MIGS are good treatment options for patients with mild-to-moderate glaucoma, those who are intolerant or noncompliant with topical eye drops, or those whose intraocular pressure (IOP) is not controllable with topical eye drops or laser trabeculoplasty [2,3].

MIGS are subdivided into three main subtypes based on the main anatomical region they focus on to improve the aqueous humor outflow and reduce the IOP: (1) trabecular meshwork bypass; (2) suprachoroidal shunts; and (3) subconjunctival filtration devices [1]. Among these categories, increasingly more ophthalmologists are choosing the subconjunctival approach as a result of promising prior reports [4,5]. The XEN gel stent and the PreserFlo MicroShunt are minimally invasive devices made to construct subconjunctival drainage routes for the aqueous humor and to effectively lower IOP in glaucoma patients. The only device among these that can be commercially used in South Korea is the XEN gel stent, which has a length of 6mm, a diameter of 45, 63, and 140 microns, and is made of a hydrophilic, porcine-derived gelatin substance that is biocompatible and flexible [6]. XEN 45 is the only form that is currently available commercially. It was conceived with the intention of increasing the safety and predictability of bleb-forming glaucoma surgical treatments [6,7].

In glaucoma surgical procedures in which filtering blebs are implanted, it is important to evaluate them postoperatively, as whether or not they are well-formed and well-functioning is an issue crucial to surgical outcomes. Previous studies have suggested several ways to categorize filtering blebs based on their clinical appearance when observed under a slit lamp [8,9]. However, such macroscopic bleb evaluation methods, including the Indiana bleb appearance grading scale (IBAGS) and Moorfield’s bleb grading system (MBGS), have limitations in identifying structural changes deeper than the conjunctiva [10,11,12].

Previous studies have reported that anterior segment optical coherence tomography (AS-OCT) is helpful for evaluating the internal morphology of filtering blebs [13,14,15,16,17,18,19]. AS-OCT imaging is a painless and non-invasive procedure facilitating quantitative measurements of internal structures in fine detail. Kawana et al. used AS-OCT to investigate the internal structures of trabeculectomy blebs, and reported that the successful blebs exhibited a large internal fluid-filled cavity, an extensive hypo-reflective area, as well as thicker walls with more microcysts [20]. However, there are few prior investigations employing AS-OCT to assess bleb morphology following XEN implantation [21,22].

Therefore, in our current study, we conducted an AS-OCT-based investigation of the internal morphology of filtration blebs after XEN gel stent implantation and determined the factors related to postoperative IOP.

## 2. Materials and Methods

A total of 18 eyes from 18 patients who had undergone XEN gel stent implantation at Seoul National University Hospital between April 2019 and December 2020 were analyzed, and the associated electronic medical records were retrospectively reviewed. This study was approved by the Seoul National University Hospital Institutional Review Board (IRB No.: 2112-116-1284). Informed consent was waived due to the study’s retrospective nature. All of the specific investigations adhered to the tenets of the Declaration of Helsinki.

### 2.1. Study Participants

This study included patients who (1) had been diagnosed with glaucoma at Seoul National University Hospital, (2) were over 18 years old, and (3) had undergone XEN gel stent implantation following an ab interno approach between April 2019 and December 2020 due to uncontrollable IOP or progression of glaucomatous change. 

Glaucoma was defined as the presence of a glaucomatous optic disc (e.g., focal notching, localized or diffuse rim thinning, or a large vertical cup-to-disc ratio), a retinal nerve fiber layer (RNFL) defect, and a glaucomatous visual field defect on standard automated perimetry (SAP) at two consecutive initial VF examinations. A glaucomatous visual field defect was defined as (1) three or more abnormal points with a probability of *p* < 0.05, of which at least one point had a pattern deviation of *p* < 0.01, or (2) a pattern standard deviation of *p* < 0.05, or (3) glaucoma hemifield test values outside of the normal limits. Each glaucoma diagnosis was confirmed by two glaucoma specialists (SW and YIS).

Patients were excluded for any of the following reasons: (1) history of intraocular surgery or laser treatment within 6 months of the XEN gel stent implantation or (2) any ophthalmic or systemic disease known to affect IOP and bleb formation. 

### 2.2. Ophthalmic Examinations

All of the subjects underwent a full ophthalmic examination, including a visual acuity (VA) assessment, slit-lamp biomicroscopy, Goldmann applanation tonometry (Haag-Streit, Koniz, Switzerland), digital color stereo disc photography, red-free RNFL photography, spectral domain optical coherence tomography (Cirrus HD-OCT, Carl Zeiss Meditec, Dublin, CA, USA), and a central 24-2 threshold test of the Humphrey visual field (HVF, HFA II; Humphrey Instruments Inc., Dublin, CA, USA). During postoperative visits, ocular complications were monitored and recorded, including hypotony, choroidal detachment, corneal decompensation, and secondary uveitis. 

### 2.3. Surgical Procedures

All of the surgical procedures were performed by two skilled surgeons (KHP and YKK) after acquiring patients’ informed consent. Following topical anesthesia, a 30-G needle was used to inject 0.1 cc of 2% lidocaine mixed with epinephrine (1:10,000, 0.1 cc) into the nasal subconjunctival region that the XEN gel stent was to occupy. Viscoelastics (DisCoVisc; Alcon Laboratories, Inc., Fort Worth, TX, USA) were injected through a 1 mm side port to maintain the anterior chamber, and then the XEN injector was guided through a 2.2 mm clear corneal incision in the temporal cornea toward the opposing superonasal target angle. Then, 3 mm from the limbus, the XEN implant was placed into the subconjunctival area. After confirming the placement of the XEN in the subconjunctival region, the injector was gently pushed backward and removed from the corneal incision. Using a surgical gonioscope, the correct positioning and length of the XEN at the target angle were confirmed. Any viscoelastic materials were removed using irrigation and aspiration. A balanced salt solution was used to hydro-seal the corneal wounds. Using a 30-G needle, 0.1 cc of mitomycin-C (MMC) 0.4 mg/mL was injected into the superonasal subconjunctival region. Topical corticosteroids and antibiotics were administered postoperatively four times daily for one month. During the follow-up, the bleb was needled under a slit lamp, and glaucoma medications were re-administered if the goal IOP could not be achieved.

### 2.4. Bleb Assessment Using AS-OCT

**Figure 1:**
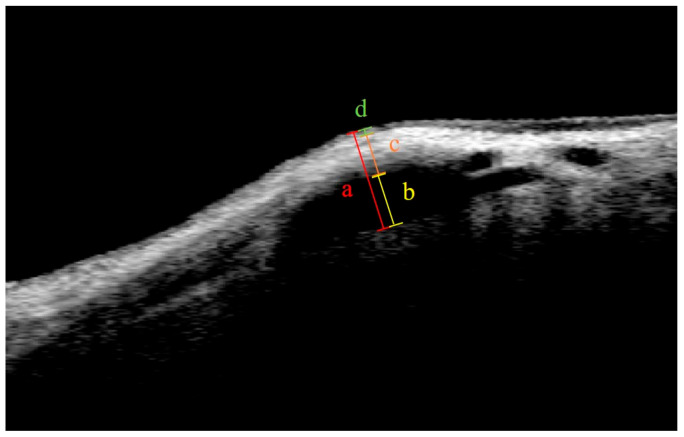
Quantitative parameters of filtration bleb: (a) bleb height; (b) height of internal cavity; (c) bleb wall thickness; (d) bleb epithelial thickness.

Blebs were imaged using Visante OCT (Carl Zeiss Meditec AG, Jena, Germany) at 6 months and 1 year after XEN gel stent implantation. The quantitative parameters measured were the maximum bleb height, the maximum height of the internal cavity, the maximum bleb-wall thickness, and the maximum bleb epithelial thickness (Figure 1). OCT calipers were used to manually measure the parameters, and the device’s software was used to automatically calculate them. Two masked glaucoma specialists (SW and YIS) independently measured the bleb parameters, and the reliability of inter-observer measurements was assessed by calculating the intraclass correlation coefficient (ICC).

### 2.5. Statistical Analysis

The baseline characteristics were compared using the independent Mann-Whitney U test for normally distributed data and analyzed using the chi-squared test for categorical data. Spearman correlation coefficients were calculated for the correlation analysis. The statistical software SPSS version 25.0 (IBM Corporation, Armonk, NY, USA) was used to perform all of the statistical analyses. *p* values of 0.05 or less were considered statistically significant.

## 3. Results

The patients’ demographics and clinical characteristics are summarized in Table 1. The bleb morphology was categorized into two groups—the internal cavity group and the uniform group—according to the presence of a definite internal cavity between the conjunctiva and sclera from AS-OCT imaging more than 6 months after surgery. The mean age was older for the internal cavity group than for the uniform group (mean ± standard deviation: 64.6 ± 13.4 vs. 51.1 ± 17.4 years; and range: 33–77 vs. 26–73 years). No significant inter-group differences were noted regarding age, sex, baseline IOP, baseline VA, baseline MD of visual field examination, number of IOP-lowering eyedrops, number of bleb needlings post-surgery, or percentage of combined cataract surgery. There were no intraoperative or postoperative complications in either group.

### 3.1. Quantitative Bleb Assessment

**Figure 2:**
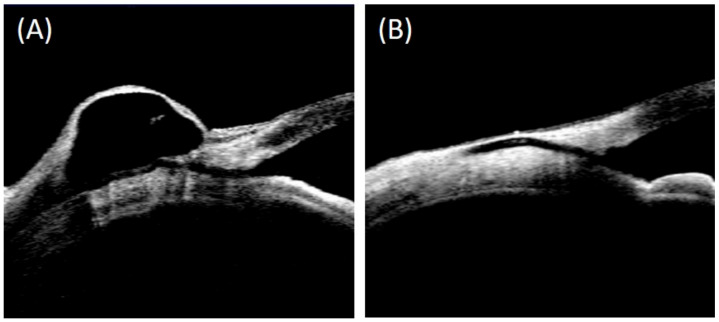
Representative AS-OCT images: (**A**) internal cavity group; (**B**) uniform group.

The quantitative parameters of the filtration bleb as measured using AS-OCT are presented in Table 2. One year following surgery, the mean bleb height was 1.28 ± 0.47 μm in the internal cavity group, and 0.70 ± 0.35 μm in the uniform group. The mean height of the internal cavity of the bleb was 0.86 ± 0.56 μm in the internal cavity group at 1 year after surgery. Bleb height and height of the internal cavity at 6 months and 1 year after surgery were both significantly higher in the internal cavity group than in the uniform group (*p* = 0.031, *p* < 0.001 and *p* = 0.039, *p* = 0.001, respectively). No significant inter-group differences were noted regarding bleb wall thickness or bleb epithelial thickness. The inter-observer agreement on the bleb parameters was excellent, with the ICC being above 0.8 in all cases (Table S1). Figure 2 (A: internal cavity group, B: uniform group) displays representative AS-OCT outcomes of patients from the internal cavity and uniform groups.

### 3.2. Comparison of Changes in IOP between Groups

**Figure 3:**
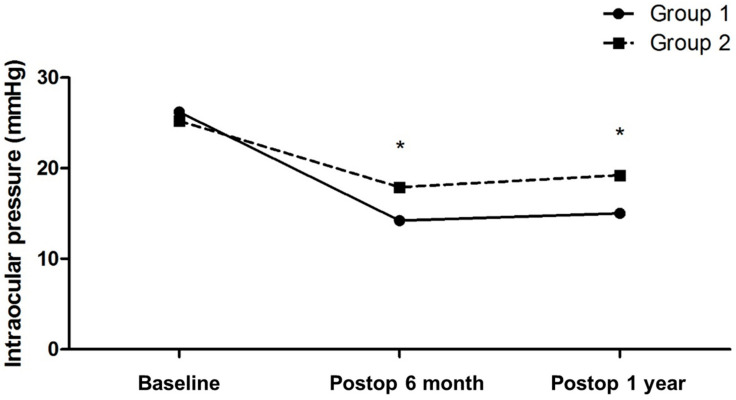
Comparison of changes in intraocular pressure (IOP) after XEN gel stent implantation between two groups. At 6 months and 1 year following surgery, IOP was significantly lower in the internal cavity group (*p* = 0.024 and *p* = 0.040, Mann-Whitney U test) than in the uniform group. Asterisks indicate a significant difference.

Changes in IOP after XEN gel stent implantation are plotted in Figure 3. IOP was measured on the same day as the AS-OCT was performed, at 6 months and 1 year after surgery. In the internal cavity group, the mean baseline IOP was 26.2 ± 7.4 mmHg, and at 6 months after surgery, it was 14.2 ± 3.0 mmHg, which showed a 43.5 ± 14.1% reduction. At 1 year after surgery, postoperative IOP was 15.0 ± 4.2 mmHg, showing a reduction of 39.1 ± 21.1% from the baseline. In the uniform group, the mean baseline IOP was 25.2 ± 5.6 mmHg and the mean postoperative IOP at 6 months and 1 year after surgery were 17.9 ± 5.0 mmHg and 19.2 ± 5.7 mmHg, respectively, demonstrating 27.9 ± 18.8% and 21.9 ± 23.9% reductions. Postoperative IOP was significantly lower in the internal cavity group than in the uniform group at both 6 months and 1 year after surgery (*p* = 0.024; *p* = 0.040).

### 3.3. Correlation between Bleb Parameters and IOP

**Figure 4:**
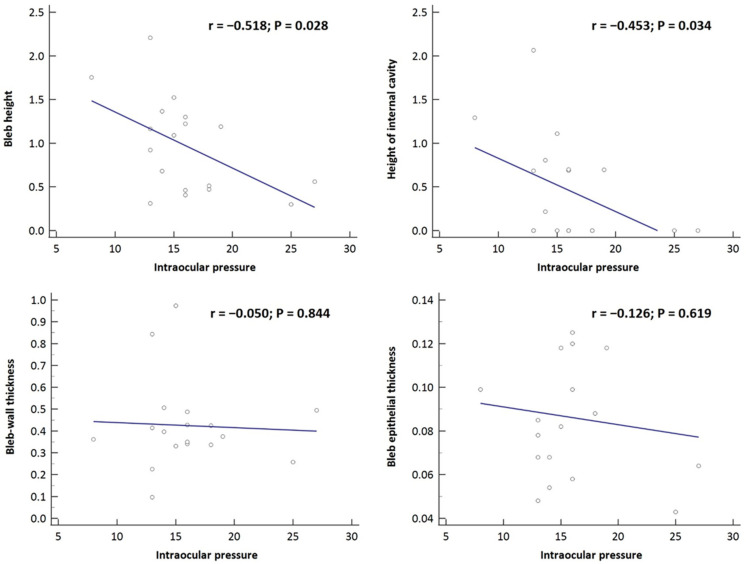
Scatterplots showing correlations between bleb parameters and postoperative intraocular pressure (IOP). Postoperative IOP showed statistically significant negative correlations with maximal bleb height and height of internal cavity (r = Spearman’s correlation coefficient).

The correlation between the quantitative bleb parameters and the baseline/mean postoperative IOP was analyzed, and the results are presented in Table 3. Baseline IOP in the internal cavity group exhibited positive correlations with bleb epithelial thickness (r = 0.781, *p* = 0.013) and negative correlations with bleb height and the height of the internal cavity (r = −0.669, *p* = 0.049 and r = −0.720, *p* = 0.029, respectively). Postoperative IOP showed statistically significant negative correlations with bleb height and the height of the internal cavity (r = −0.518, *p* = 0.028 and r = −0.453, *p* = 0.034, respectively; Figure 4). Postoperative IOP did not show any statistically significant correlation with bleb wall thickness, or bleb epithelial thickness (*p* = 0.844; *p* = 0.619).

### 3.4. Correlation between Bleb Parameters and Clinical Characteristics

Table 4 displays the findings from an analysis of the relationship between clinical features and quantitative bleb parameters. Age and bleb height had a positive correlation (r = 0.561, *p* = 0.015). There were negative correlations between the amount of glaucoma medication taken at baseline and the bleb height (r = −0.722, *p* = 0.028 in internal cavity group; r = −0.486, *p* = 0.041 in entire population). The duration of the glaucoma and sex did not significantly affect the bleb parameters.

## 4. Discussion

In this retrospective observational study, we evaluated filtration bleb morphology after XEN gel stent implantation using AS-OCT and investigated the factors related to IOP postoperatively. After surgery, IOP was significantly lower in patients with internal bleb cavities, and an increased height of the internal cavity appeared to be associated with a lower IOP. 

The XEN gel stent is a relatively novel device in glaucoma surgery, and prior studies have shown encouraging performance data [23,24,25,26,27]. It is a hydrophilic tube made of porcine gel that has been cross-linked with glutaraldehyde for good stability and biocompatibility with little tissue reaction [28]. It is of 6 mm length and 45 μm lumen size, and is typically implanted into the subconjunctival space 3 mm from the limbus. Therefore, the XEN bleb is positioned more posteriorly at the beginning and is of a flatter shape compared with blebs created using trabeculectomy. According to previous studies, XEN blebs are significantly flatter than trabeculectomy blebs [29,30].

Nakano et al. reported that 20.8% of blebs after trabeculectomy could be classified as uniform blebs, showing no fluid cavities or spaces in the conjunctiva [31]. According to Lenzhofer et al., implantation of the XEN gel stent resulted in a higher rate of uniform blebs (48%) than did trabeculectomy [32]. Our study, meanwhile, showed a similar rate of uniform blebs (50%). These differences in bleb distribution between trabeculectomy and XEN gel stent implantation might be explained by differences in the degrees of conjunctival trauma and wound healing between the two surgeries. 

Lenzhofer et al. investigated the internal bleb morphology over time using AS-OCT, on which basis they posited a correlation between IOP and surgical long-term success with bleb morphology. They suggested that the presence of tiny, dispersed cysts was directly associated with a lower IOP, whereas cystic encapsulation after 3 months was related to a higher risk of surgical failure [32]. In the present study, blebs with an internal cavity showed lower postoperative IOP, and height of internal cavity was associated with a lower postoperative IOP. Diffuse subconjunctival cysts or an internal cavity may give blebs a greater internal capability to regulate aqueous humor outflow. 

The relationships between bleb parameters and patients’ clinical characteristics were also examined in this study. Age and amount of glaucoma medication taken before surgery both showed a strong positive correlation with the bleb height. The association between patient’s age and the surgical success rate is still a matter of debate. According to some research, a thicker Tenon’s capsule and younger patients’ more active healing processes are probably the cause of the poor success rates in such individuals [33,34]. Other studies, however, contend that application of MMC results in filtration blebs that are thinner, avascular, and more hypocellular with increased atrophic stroma can improve surgical outcomes by inhibiting fibroblast proliferation of conjunctiva and preventing scarring [34,35]. In order to demonstrate the impact of age, we therefore need more longitudinal observational studies on large populations. Previous studies have shown that a substantial risk factor for failure of the filtering operation is the cumulative number of topical anti-glaucoma medication [36,37,38], and this is consistent with our results. Conjunctival goblet cells are reduced in eyes undergoing long-term topical glaucoma treatment, while fibroblasts, macrophages, and lymphocytes are increased [39,40]. Furthermore, conjunctival metaplasia is associated with the quantity of glaucoma eye-drops [41]. This may result in conjunctival scarring, which has been connected to lower success rates of filtration surgery [38]. This study demonstrated that such abnormalities in the conjunctiva apply to MIGS as well as traditional glaucoma surgery.

In this study, the surgeon injected 0.1 cc of MMC 0.4 mg/mL into the superonasal subconjunctival space after implantation of the XEN gel stent. According to Seol et al., the MMC concentration has no effect on the trabeculectomy blebs’ vascularity or height [42]. However, hypotony is relatively prevalent in the early postoperative period following XEN implantation, and appears to be triggered by the peritubular flow produced by the difference in diameter between the XEN gel stent and the injector needle carrying it [6,25]. In our study nonetheless, there was no case of hypotony. This may be because all of the XEN gel stents were injected into the subconjunctival space more than 3 mm from the limbus and that all of the XEN gel stents were successfully injected the first time rather than requiring several attempts, which could have reduced peritubular flow.

Our study has several limitations. First, all of the patients were Korean, and thus, additional research with populations from other racial and ethnic groups will be required to confirm our findings. Second, the small number of study subjects may have influenced the analysis and interpretation of the results. Because we included only patients who had undergone XEN implantation exclusively utilizing the ab interno procedure and excluded patients with insufficient follow-up visits, our study can be considered to be limited in the number of its participants. Further studies analyzing a larger number of eyes are warranted. Third, needling, which is a frequently performed procedure after filtering surgery, can affect bleb morphology. Since most needling in our case was performed within the immediate postoperative period, we performed bleb evaluations more than 6 months after surgery in order to minimize the potential effect of needling.

## 5. Conclusions

In conclusion, AS-OCT facilitates the analysis of bleb morphology after XEN gel stent implantation. A larger height of the internal cavity of the bleb correlates with a lower IOP after XEN gel stent implantation. Knowing the relationships between a bleb’s morphological characteristics and postoperative IOP, where the latter is directly associated with the surgical outcome, would be very helpful for patient monitoring and management following XEN gel stent implantation.

## Figures and Tables

**Table 1:** Demographics and clinical characteristics of study subjects.

	Internal-Cavity	Uniform	*p* Value
Number of eyes	9	9	
Age (years)	64.6 ± 13.4	51.1 ± 17.4	0.077 ^a^
Sex (M:F)	3:6	5:4	0.343 ^b^
Baseline IOP (mmHg)	24.1 ± 5.6	24.6 ± 6.5	0.931 ^a^
Baseline VA (logMAR)	0.63 ± 0.91	0.67 ± 1.08	0.340 ^a^
Baseline MD (dB)	−20.06 ± 8.81	−11.66 ± 9.07	0.142 ^a^
Number of anti-glaucoma medications			
Baseline	3.6 ± 0.7	3.8 ± 0.8	0.546 ^a^
6 months after surgery	1.4 ± 1.2	2.3 ± 1.4	0.612 ^a^
1 year after surgery	1.9 ± 1.2	2.1 ± 1.1	0.238 ^a^
Number of bleb needlings	0.3 ± 0.5	0.6 ± 1.1	0.198 ^a^
Combined cataract surgery (%)	11.1	22.2	0.527 ^b^

Values are mean ± standard deviation. ^a^ Mann-Whitney U test, ^b^ Chi-square test. IOP, intraocular pressure; VA, visual acuity; MD, mean deviation.

**Table 2:** Quantitative parameters of filtration bleb.

	Internal Cavity	Uniform	*p* Value
6 month post-surgery			
Bleb height (μm)	1.23 ± 0.55	0.69 ± 0.32	**0.031 ^a^**
Height of internal cavity (μm)	0.74 ± 0.58	0.00 ± 0.00	**<0.001 ^a^**
Bleb wall thickness (μm)	0.38 ± 0.12	0.47 ± 0.26	0.340 ^a^
Bleb epithelial thickness (μm)	0.10 ± 0.03	0.10 ± 0.02	0.931 ^a^
1 year post-surgery			
Bleb height (μm)	1.28 ± 0.47	0.70 ± 0.35	**0.039 ^a^**
Height of internal cavity (μm)	0.86 ± 0.56	0.00 ± 0.00	**0.001 ^a^**
Bleb wall thickness (μm)	0.30 ± 0.24	0.41 ± 0.42	0.495 ^a^
Bleb epithelial thickness (μm)	0.08 ± 0.02	0.08 ± 0.04	0.755 ^a^

Values are mean ± standard deviation. ^a^ Mann-Whitney U test. Bold values indicate that *p* values were statistically significant (*p* < 0.05).

**Table 3:** Correlations between bleb parameters and postoperative intraocular pressure.

Baseline Intraocular Pressure						
	Internal Cavity		Uniform		Total	
	r	*p*	r	*p*	r	*p*
Bleb height (μm)	−0.669	**0.049**	−0.017	0.966	−0.253	0.310
Height of internal cavity (μm)	−0.720	**0.029**	N/A	N/A	−0.245	0.327
Bleb wall thickness (μm)	0.452	0.222	−0.042	0.915	0.245	0.327
Bleb epithelial thickness (μm)	0.781	**0.013**	−0.025	0.949	0.343	0.163
**Postoperative Intraocular Pressure**						
	**Internal Cavity**		**Uniform**		**Total**	
	r	*p*	r	*p*	r	*p*
Bleb height (μm)	−0.405	0.279	−0.324	0.395	−0.518	**0.028**
Height of internal cavity (μm)	−0.396	0.292	−0.204	0.661	−0.453	**0.034**
Bleb wall thickness (μm)	0.209	0.589	−0.280	0.465	−0.050	0.844
Bleb epithelial thickness (μm)	0.368	0.330	−0.454	0.220	−0.126	0.619

r = Spearman’s correlation coefficient; *p* = *p*-value. Bold values indicate that *p* valued were statistically significant (*p* < 0.05).

**Table 4:** Correlations between bleb parameters and clinical characteristics.

Age						
	Internal Cavity		Uniform		Total	
	r	*p*	r	*p*	r	*p*
Bleb height (μm)	0.102	0.795	0.567	0.112	0.561	**0.015**
Height of internal cavity (μm)	0.000	1.000	N/A	N/A	0.398	0.102
Bleb wall thickness (μm)	0.599	0.117	0.400	0.286	0.416	0.086
Bleb epithelial thickness (μm)	−0.094	0.810	0.650	0.058	0.217	0.387
**Male Sex**						
	**Internal Cavity**		**Uniform**		**Total**	
	r	*p*	r	*p*	r	*p*
Bleb height (μm)	0.091	0.815	−0.520	0.152	−0.280	0.260
Height of internal cavity (μm)	0.000	1.000	N/A	N/A	−0.207	0.409
Bleb wall thickness (μm)	0.183	0.638	−0.346	0.361	−0.194	0.441
Bleb epithelial thickness (μm)	0.506	0.164	−0.173	0.656	0.173	0.493
**Duration of Glaucoma**						
	**Internal Cavity**		**Uniform**		**Total**	
	r	*p*	r	*p*	r	*p*
Bleb height (μm)	−0.500	0.170	0.067	0.865	−0.178	0.481
Height of internal cavity (μm)	−0.383	0.308	N/A	N/A	−0.207	0.409
Bleb wall thickness (μm)	0.550	0.125	0.117	0.765	0.240	0.338
Bleb epithelial thickness (μm)	−0.160	0.682	−0.100	0.798	−0.095	0.709
**Amount of Anti-Glaucoma Medication at Baseline**						
	**Internal Cavity**		**Uniform**		**Total**	
	r	*p*	r	*p*	r	*p*
Bleb height (μm)	−0.722	**0.028**	−0.298	0.436	−0.486	**0.041**
Height of internal cavity (μm)	−0.552	0.123	N/A	N/A	−0.363	0.138
Bleb wall thickness (μm)	0.027	0.946	−0.056	0.886	−0.085	0.737
Bleb epithelial thickness (μm)	0.094	0.809	−0.093	0.812	0.000	1.000

r = Spearman’s correlation coefficient; *p* = *p*-value. Bold values indicate that *p* values were statistically significant (*p* < 0.05).

## Data Availability

The data in this study are available on reasonable request from the corresponding author.

## Supplementary Materials

The following supporting information can be downloaded at: https://www.mdpi.com/article/10.3390/jcm12216740/s1, Table S1: Inter-observer agreement for bleb parameters.
