# Supplementary material for: Bleb Morphology on Anterior-Segment Optical Coherence Tomography after XEN Gel Stent Implantation
Source: J Clin Med. 2023 Oct 25;12(21):6740. doi: 10.3390/jcm12216740 (PMC10648008; doi:10.3390/jcm12216740)
Supplement: Supplementary file 1 [file jcm-12-06740-s001.zip › jcm-2650603-supplementary.pdf]

Table S1. Inter-observer agreement for bleb parameters

	Intraclass correlation coefficient	95% CI	
		Lower limit	Upper limit
6-month post-surgery			
Bleb height	0.998	0.996	0.999
Height of internal cavity	0.982	0.951	0.993
Bleb-wall thickness	0.906	0.752	0.964
Bleb epithelial thickness	0.805	0.689	0.968
1-year post-surgery			
Bleb height	0.996	0.988	0.999
Height of internal cavity	0.981	0.962	0.996
Bleb-wall thickness	0.989	0.968	0.997
Bleb epithelial thickness	0.880	0.804	0.927

CI: confidence interval
